# Deployment-related mental health support: comparative analysis of NATO and allied ISAF partners

**DOI:** 10.3402/ejpt.v5.23732

**Published:** 2014-08-14

**Authors:** Eric Vermetten, Neil Greenberg, Manon A. Boeschoten, Roos Delahaije, Rakesh Jetly, Carl A. Castro, Alexander C. McFarlane

**Affiliations:** 1 Military Mental Health Research, Department of Defence, Utrecht, The Netherlands;; 2 Department Psychiatry, Leiden University Medical Center, Leiden, The Netherlands;; 3 Arq Psychotrauma Research Group, Diemen, The Netherlands; 4 Academic Centre for Defence Mental Health, Weston Education Centre, Kings College London, London, UK; 5 Netherlands Organization for Applied Scientific Research (TNO), Defense Safety and Security, Soesterberg, The Netherlands; 6 Directorate of Mental Health, Health Services Group, National Defence, Ottawa, Canada; 7 School of Social Work, University Southern California, Los Angeles CA, USA; 8 Centre for Traumatic Stress Studies, University Adelaide, Adelaide, Australia

**Keywords:** Military, deployment, mental health, NATO, review

## Abstract

**Background:**

For years there has been a tremendous gap in our understanding of the mental health effects of deployment and the efforts by military forces at trying to minimize or mitigate these. Many military forces have recently systematized the mental support that is provided to support operational deployments. However, the rationale for doing so and the consequential allocation of resources are felt to vary considerably across North Atlantic Treaty Organisation (NATO) International Security Assistance (ISAF) partners. This review aims to compare the organization and practice of mental support by five partnering countries in the recent deployment in Afghanistan in order to identify and compare the key methods and structures for delivering mental health support, describe bottlenecks and illustrate new developments.

**Method:**

Information was collected through document analysis and semi-structured interviews with key military mental healthcare stakeholders. The review resulted from close collaboration between key military mental healthcare professionals within the Australian Defense Forces (ADF), Canadian Armed Forces (CAF), United Kingdom Armed Forces (UK), Netherlands Armed Forces (NLD), and the United States Army (US). Key stakeholders were interviewed about the mental health support provided during a serviceperson's military career. The main items discussed were training, prevention, early identification, intervention, and aftercare in the field of mental health.

**Results:**

All forces reported that much attention was paid to mental health during the individual's military career, including deployment. In doing so there was much overlap between the rationale and applied methods. The main method of providing support was through training and education. The educative focus was to strengthen the mental resilience of individual soldiers while providing a range of mental healthcare services. All forces had abandoned standard psychological debriefing after critical incidents. Instead, by default, mental healthcare professionals acted to support the leader and peer led “after action” reviews. All countries provided professional mental support close to the front line, aimed at early detection and early return to normal activities within the unit. All countries deployed a mental health support team that consisted of a range of mental health staff including psychiatrists, psychologists, social workers, mental health nurses, and chaplains. There was no overall consensus in the allocation of mental health disciplines in theatre. All countries (except the US) provided troops with a third location decompression (TLD) stop after deployment, which aimed to recognize what the deployed units had been through and to prepare them for transition home. The US conducted in-garrison ‘decompression’, or ‘reintegration training’ in the US, with a similiar focus to TLD. All had a reasonably comparable infrastructure in the field of mental healthcare. Shared bottlenecks across countries included perceived stigma and barriers to care around mental health problems as well as the need for improving the awareness and recognition of mental health problems among service members.

**Conclusion:**

This analysis demonstrated that in all five partners state-of-the-art preventative mental healthcare was included in the last deployment in Afghanistan, including a positive approach towards strengthening the mental resilience, a focus on self-regulatory skills and self-empowerment, and several initiatives that were well-integrated in a military context. These initiatives were partly/completely implemented by the military/colleagues/supervisors and applicable during several phases of the deployment cycle. Important new developments in operational mental health support are recognition of the role of social leadership and enhancement of operational peer support. This requires awareness of mental problems that will contribute to reduction of the barriers to care in case of problems. Finally, comparing mental health support services across countries can contribute to optimal preparation for the challenges of military deployment.

Deployment is an important part of every military career and most service members return from deployment with a sense of satisfaction. The nature, objective, and operations tempo (OPSTEMPO) of military missions has been highly variable during the last century; however, most service personnel typically participate in a series of deployments in their military career. This OPSTEMPO as well as the burden of working in dangerous situations places demands on behavioral or mental healthcare. Yet, despite improvements in pre-deployment training and preparation over recent years, some service members will return from deployment with some cost to their health. The results can manifest in terms of behavioral/physical and mental health (MH) problems, as has been spelled out in various studies in relation to the most recent deployments (Creamer, Burgess, & McFarlane, 2001; Engelhard et al., 2007; Fear et al., 2010; Hoge, Auchterlonie, & Milliken, 2006; Hoge et al., 2008; Killgore et al., 2008; Vasterling et al., 2010; Wittchen et al., 2012). The prevalence of persistent disrupted sleep, headaches, fatigue, or symptoms associated with other stresses and combat-related disorders such as posttraumatic stress disorder (PTSD), traumatic brain injury (TBI), depression, or ill-defined health conditions may vary across nations (Iversen et al., 2009; Kelsall et al., 2009; Kok, Herrell, Thomas, & Hoge, 2012; Luxton et al., 2011; Sareen et al., 2007; Smith et al., 2009; Theeler, Mercer, & Erickson, 2008; Thomas et al., 2010) but are not uncommon in any of them.

Most military organizations have professionalized behavioral and MH care they provide, in parallel to their medical care. North Atlantic Treaty Organisation (NATO) partners face similar challenges in deployment situations such as in Afghanistan, and thus increasingly favor collaboration across countries in current behavioral and MH practices. Recent conflicts in Iraq and Afghanistan have led to the deployment of military (field) hospitals which are equipped with material and specialists, and in tandem military MH services and disciplines operate in the deployed environment to deliver care for the psychologically injured soldier and to enact robust preventative mental healthcare. Several nations have started strategies to help service personnel overcome the stigma associated with seeking psychological help and encourage appropriate help-seeking (Adler et al., 2013).

The goals of this study were to: (1) assess existing protocols and current practices of MH support before, during and after operational deployment, focusing on prevention, intervention, and treatment; (2) provide a comparative analysis of existing protocols and current practices; and (3) identify common bottlenecks for effective MH support and promising future developments. The focus was especially targeted on current practices and new developments with regard to training programs, interventions, and treatment procedures of military organization during and after deployment. However, when service personnel are redeployed frequently MH support after deployment becomes pre-deployment MH support. Therefore, it was considered that MH support after deployment should be considered an integrated part of the whole chain of MH support within the military organization. Hence, for the purpose of this analysis we focused on the complete chain of MH support, which included: (1) stress management training (general education of military personnel); (2) readiness training before deployment; (3) general MH support in-theatre; (4) interventions after a potentially traumatic incident in-theatre; (5) decompression, and (6) post-deployment MH support (at home).

## Method

Information was collected in 2010 by document analysis and by interviewing key-stakeholders in the field of MH support of each nation. Information was gathered on current practices of MH service of five different countries, all serving with major troop contributions in the recent deployment in Afghanistan. The countries that were selected in this project were Australia (AM, AUS), Canada (RJ, CAN), Great Britain (NG, GBR), the United States of America (CC, US). Information from the Netherlands was performed through several leading commanders. The chosen nations in this study have shared interests, concerns, and needs because they were all active in the NATO-ISAF mission in Afghanistan and the service personnel of these nations were all exposed to similar occupational hazards. We were aware that there are more nations (whether active in Afghanistan or not) that have similar needs. These nations were not excluded on any formal grounds; we included the main players with the largest contribution to the Afghanistan operations.

First, a semi-structured interview protocol, constructed using a Delphi method with the help of key leaders within the NLD Armed Forces, was used for both choosing the relevant topics for the interviews and document analysis. Next, two levels of data-acquisition were initiated. One was during formal meetings with MH experts of the different nations, during public presentations and in one-on-one interviews for a duration of 1–2 hours in parallel to this. The second level was through a formal request to the Surgeon Generals of the participating nations to review unclassified reports and existing documentation. Both document analysis and interviews were focused on the MH organization of the partner's Afghanistan mission as ISAF between the years 2008 and 2010. The Delhi method resulted in the identification of six main topic areas to be assessed: (1) policies and current practices regarding MH support and stress management interventions; (2) underlying (scientific, cultural, logistic, or otherwise-based) rationale behind these policies and practices; (3) operational procedures after the occurrence of a traumatic incidents (e.g., improvised explosive devices [IEDs]); (4) operational procedures after deployment; (4) policy related to OPSTEMPO; (5) evaluation of these policies, operational procedures, and interventions; and (6) future developments regarding policies, procedures, and interventions (the semi-structured interview format is available upon request).

## Results

**Fig. 1:**
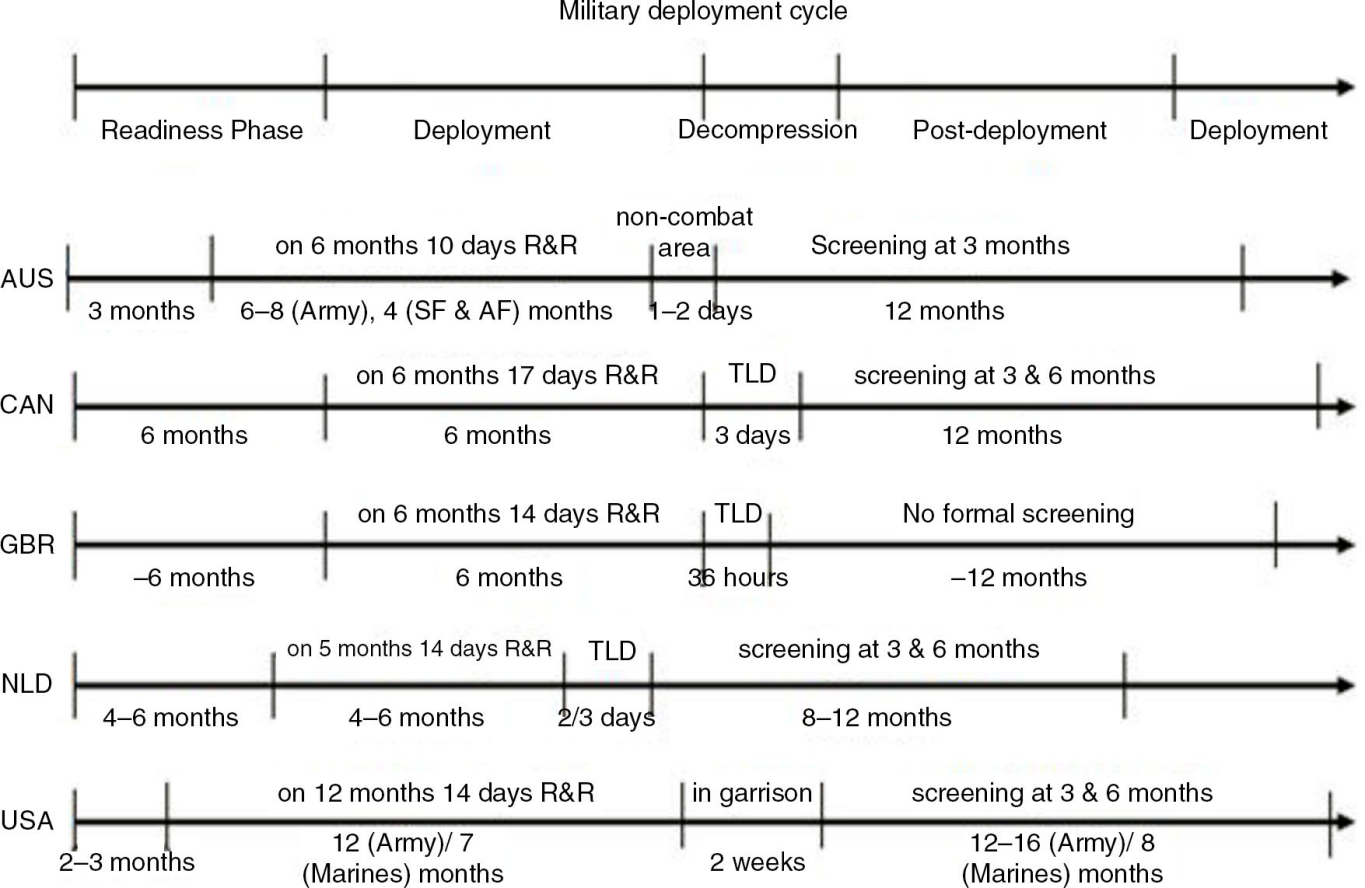
The military deployment-cycle time-line of AUS, CAN, GBR, NLD, and USA for the NATO-ISAF Mission in Afghanistan. This time-line is considered of interest, as it probably significantly influences the MH-cycle of service members; that is, going from being in balance/resilient after pre-deployment training, to getting injured by stress in-theatre, back to becoming in balance again during R&R or decompression.

The current report describes the results of both the document-review and semi-structured interviews. The MH protocols and practices of the five different NATO-partners are reported here. The deployment cycle is illustrated in Fig. 1. Table 1 illustrates the mission, unit, and MH characteristics across the participating nations. The comparison was descriptive and more detailed comparison was not in the scope of this study. One of the challenges in describing these interventions is the low quality of systematic evidence about their effectiveness (Institute of Medicine [IOM], 2014). This analysis provides a qualitative outline of these programs to assist in their further refinement and investigation.

### MH practices during the pre-deployment phase

#### Deployment-specific MH health care plan

Most of the participating partners underscored that MH risk and needs assessment for each substantial deployment was essential for success of the operation. The risks to personnel and the possible mental health impact vary from peacekeeper to combat missions. This required a tailored program to be put in place that was based on the outcome of the risk assessment. For example, the hazards in some missions need to focus more on dealing with extremes of suffering and dealing with mass graves than the immediate strains of combat. The advantage of this practice was that the required training for service personnel, command line, and the MH support team could be tailored to the specific mission, to enhance preventative effects. Also, the type and number of MH professionals could be adjusted to the specific mission, which aimed to ensure early detection and treatment for emergent MH problems among service personnel. Nations intended that carrying out a pre-deployment mission-specific MH risk and needs assessment would limit “surprises” during a mission by ensuring that proper MH measures were in place. Nevertheless, undertaking such an assessment and doing various adjustments placed an additional load on the general MH support system of a military organization. If the general MH support system did not have enough resources to carry out the risk assessment, the collective impression was that it might be wiser not to undertake this effort since it could diminish the quality of the MH support actually delivered. Instead, when resources in the system were limited it was considered wiser to have a flexible approach and adjust MH support on the basis of risks and needs. An option was to fly in an MH support team for care or for post-mission MH training and screening.

**Table 1:** The mission, unit and MH characteristics of AUS, CAN, GBR, NLD, and USA for the NATO-ISAF mission in Afghanistan

Mission specifics					Unit specifics		

	Length	Interval between missions	Number soldiers deployed	Scheduled R&R	Unit demographics	Length of time together before deployment	Continuity of unit (new members, life time)
AUS	Special Operations Command (SOC) = 4 mo. Regular Army 6 to 8 mo. RAAF Aircrew 4 mo. RAAF ground staff—6 mo. Navy—approx 6 mo.	SOC and aircrew can do roughly 4 months per calendar year. Minimal interval for all others is 1 year.	~2,350 service members in Afghanistan. ADF total full time staff is ~ 55,000.	For missions 6 mo. and over, members get 10 days ROCL available from the half-way point of tour through to last mo. of tour.	Units consist of mainly RF but also some Reserve members. Age, deployment experience and background varies between members. Depending on the type of task of the unit, gender may be balanced or biased toward males.	Variable but formed units usually together for a fair while (mo. to yrs). SF teams usually meet up 3 mo. before and do some pre-mission training with the Dutch.	RF units are relatively stable, i.e., unit members are often together for quite some time before they leave on deployment and they stay together for multiple operations. However, SF teams are formed ad hoc for a certain task and will also be taken apart afterwards. Due to this, SF teams have a shorter lifetime.
CAN	In 95% of the cases service members will be deployed for 6 months. (Medical) specialists are deployed shorter, i.e., 3 mo. Commanders can be deployed for 1 year.	The min interval between missions is 1 year. If service members volunteer to go on next mission earlier, they sign a waiver. In practice, the interval varies between 18 and 24 months.	~2,500 service members in Afghanistan. CF has ~ 70,000 RF and 30,000 Reserve Force members.	There is mid-tour scheduled R&R. In practice, this will be between the 1st and 4th month during deployment. Service members are 17 days away from the mission area.	Units consist of both Regular Force and Reserve members. Age, deployment experience and background varies between members. Depending on the type of task of the unit, gender may be balanced or biased toward males.	Regular Force unit members are together for a long time, often > year. Reserve unit members are usually added later. However, the whole unit is together before deployment at least 6 months (during pre-deployment training).	The aim is to have a long unit life time (i.e., multiple deployments with same unit). Nevertheless, augmentation of units by Reserve members does occur.
GBR	Six months as standard. Some less than this (e.g., specialist medical personnel), some HQ personnel do 12 months.	Guidelines state no more than 12 months deployed in any 3-year period.	~9,000 in Afghanistan. UK AF has about ~190,000 RF and ~87,000 Reserve members.	14 days per 6 months allowed—which should allow 10 days at home.	Very varied—all types of units and specialist teams are deployed.	Varies—the main combat units are formed anyhow and IR (individual reinforcements) join such units a few months before deployment. Generally a 6 month reservist's tour would mean they were mobilized for about a year.	Personnel move between units every 2–3 years. Generally non-officers stay within the same regimental system (1–5 Battalions per regiment) and officers alternate between regimental and other postings. However, the postings schedule vary considerably.
NLD	Four or six months: depends on task (Battlegroup=4, Task Force=6).	At minimum twice the time of earlier deployment.	~1,200 in Afghanistan. NLD Army has ~ 52.000 members.	For missions longer than 5 months personnel get approx 2 weeks leave at home.	Unit consists of regular force. Varies in age and experience. Depending on type of task more males.	There is a mission specific preparation program for approx 4–6 months. Ideally, before this time unit should be formed, but this is not always possible.	Personnel change position every 3 years. In addition, after deployment some service members leave military and thus unit will receive new members.
USA	Typically 12 months for Army. 7 months for Marines, 4–6 months for SF.	Typically 12–16 months for Army, 8 months for Marines, 4–6 months for SF.	60,000 in Afghanistan. In total the US AF consist of 1,473,900 active personnel and 1,485,500 reserve personnel.	One must be deployed 12 months to qualify for 14 days mid-tour leave. With deployments of 15 months it is 17 days.	For Army units comprise the entire spectrum from combat, service support to combat service support, plus special operations. Age, deployment experience, and background varies between members.	Highly variable. Can range from years to weeks. Movement out of a unit stops approx. 2–3 months before deployment so most Soldiers are together for several months prior to deploying, but there are last minute fills, so Soldiers can be very new to the unit.	Personnel move about every 3 years or so.
MH support in pre-deployment phase					MH support in deployment phase			

	Mission-specific MH care plan	MH screening in service members	MH education/training in service members (which topics & delivered by whom?)	MH team available (which members?)	Type of MH support provided by MH team (type of screening/de-briefing/therapies used)	Type of MH support provided by own unit (by commander/by buddies)	Repatriation (when, who decides & how?)

AUS	Although the ADF tries to identify mission-specific MH threats they do not create a mission-specific MH care plan.	The ADF do not undertake pre-deployment MH screening. Instead, the ADF work with a Medical Employment Classification system to assess whether service members are able to deploy or not. Also, the results of post-deployment MH assessments of the last deployment are used (RtAPs and POPS).	All given by Directorate of MH. Resilience and pre-deployment training (recently introduced BattleSMART Self-Management and Resilience Training program) and a pre-deployment briefing by a psychologist.	MO, psychological examiner, a chaplain and a psychologist. No SWs.	No standard in-theatre MH screening or debriefing. CO does operational debriefs. Self-referral or by CO to MH team. MH team can provide MH first aid. For more formal treatment ADF relies on MH professionals of NATO partners or repatriation follows.	Padre's—TLC Mates—informal debriefs, buddy support Chain of command—formal debriefs, advice.	MO usually in consult with CO.
CAN	A mission-specific MH threat assessment is carried out to determine the type of MH team that should join the unit. This is based on # service members deployed and exposures they could experience. Also, assessed is whether additional training is required (i.e., as an augmentation to standard readiness training).	There is two-fold MH screening: MH inquiries are done during annual physical. Also, pre-deployment, each service member is seen by a MO who gives a “rating” for deployability (green, yellow or red). Moreover, a service member is seen by an MH nurse/SW, who focuses on family support plan. Both advise commander who customarily follows this combined advice.	First, there is MH education throughout the carrier by the MH & Operational Stress Injury Joint Speakers Bureau (MH & OSI JBS). It is focused on increasing MH and OSI literacy, while targeting attitudes and stigma around MH. Secondly, there is “Road to Mental Readiness” (R2MR) training before a mission. It is focused on preparation for and mitigation of the stresses of operations and deployment. A team of MH professionals and trained peers delivers both types of training, but in the delivery the units’ own commander takes central role.	During current mission multiple MH nurses and SWs are available and at least one psychiatrist. Also there are chaplains available. CF do not have uniformed psychologist, but can reply on uniformed psychologist of other NATO partner, if needed.	No in-theatre MH screening. Service members may self-refer to whom they want (no barrier to referral). Usually, MH nurse/SW does 1st assessment and refers to psychiatrist if needed. MH nurse/SW focuses on family matters and psychosocial issues. Psychiatrist focuses on formal diagnoses and treatment. Case management is always coordinated between commander and MH team. Therapy is usually CBT, but may also be EMDR or medication. There is no standard critical incident debriefing. However, if decided necessary by the commander and MO a tailor-made brief is given.	During the MH & OSI JBS carrier courses and R2MR training units are taught about MH and OSI awareness, recognition of common behavioral signs of MH issues and OSIs and supportive buddy/leadership skills and actions. Commanders work closely with MO and MH team to support their unit and provide a work environment that is conductive to positive coping and MH.	Repatriation is ultimately the decision of commander again in coordination with MOs. This decision is based on severity of illness, individual's response to treatment, specific job, MH risks of staying versus MH risks of leaving unit. Aim is to keep individual with unit as long as possible since this is often more advantageous for individuals MH.
GBR	No mission-specific MH care plan. However, it is acknowledged that mission demands may vary for the different Services. Therefore, each Service has a Consultant Advisor in Psychiatry who advises regarding service-specific MH requirements and policy.	None formally. Does not work. Unit medical and welfare staff discuss risky cases with commanders and make decisions.	All personnel should receive an MH brief prior to deployment and another short one in theatre. Briefs given by medical, MH or TRiM personnel. May include body-handling information where appropriate for tasking.	Field MH Team (FMHT) consists of three psychiatric nurses (at least one of which is an officer) and a visiting psychiatrist every 3 months—visits last about 10 days.	No in-theatre screening or debriefing. MH support consists of liaison, formal treatment and TRiM support.	Buddy Aid, TRiM, Padres (in some locations) and most units have some medical personnel who have varying degrees of MH training.	Final decision lies with MOs or FMHT.
NLD	A mission-specific MH plan is made on basis of needs and risk assessment. The plan indicates training needs and needs for MH support in theatre.	No official screening. Unit commanders and social medical team of unit discuss deployability of service members.	All personnel attend pre-deployment stress management briefings given by psychologist and SW. Additional training can be requested by commander.	The Social Medical Team (SMT) consists of a MO, chaplain, SW and psychologist. Psychiatrists are not deployed.	No standard screening. No standard debrief by MH professionals, but MH professionals are often present at operational debrief. SW focus on psychosocial problems. Psychologist focus on psychological problems and provide treatment (CBT, EMDR, etc.).	Unit members and chaplain provide informal social support. Commander leads formal debriefs.	Final decision lies with commander. MH professionals (SMT) advise.
USA	The unit MH team conducts a unit risk assessment. Besides unit based MH support, area based MH support is provided when necessary for a mission. For this, an area support needs assessment is conducted based on troop strength, location, mission.	There is no official pre-deployment screening to assess fitness for deployment. All medical records are reviewed by the Brigade MO to ensure medical fitness for deployment.	Army receives pre-deployment Battlemind which focuses on the expectations of combat and effective coping skills that soldiers and leaders can employ.	An extremely robust cadre of MH providers support the deployed force, including organic MH assets and Combat Stress Control teams.	No standard debriefing by MH personnel, but commander can request an event-based Battlemind psychological debriefing. Treatment: the entire spectrum, from unit MH needs assessment to treatment and restoration to command consultation.	Self-aid, buddy aid. Chaplains provide spiritual support/counseling. Commanders/leaders can request Combat Stress Control support as well.	In case of serious MH problems, MH professionals advise the commander on repatriation. However, the goal is to “restore” in proximity of the unit. For this restoration the Combat Stress Control Unit provides facilities.
MH support in post-deployment phase							

	TLD(how long, what main elements)	Follow-up and care by MH professionals (screening, treatment, etc.)		Follow-up and care by unit		MH services infrastructure (clinics, networks, programs)	

AUS	None currently—maybe one day on way out due to travel delays, but see RtAPs in next column.	RtAPs (in non-combat area) before leaving country; POPs at 3 month post-deployment. RtAPS consists of three main parts: – a group debrief on return from deployment issues, – psychological screening, – interview with psychologist/psychological examiner. When a referral is needed command line is notified. It depends on the screening output whether a psychologist or psychological examiner conducts the interview. POPS consists of 2 main parts: • self-report MH screening conducted by a psychologist, • interview with either psychologist/psychological examiner to conduct a more in-depth screening, and to address adjustment issues, and provide information. Service members who are encountering MH problems are referred for counseling.		Nothing formal—COs and mates; buddy support		Up to 2009, the DMH used Regional MH Teams (RMHT) to obtain its goals: These are present in places where there are large concentrations of service members. RMHT are multi-disciplinary bodies comprised of representatives from the range of ADF MH services. RMHT promote treatment programs, manage complex cases, coordinate local networks, provide outpatient care, deliver critical incident MH support on demand and coordinate prevention strategies/programs. At unit level, MH support is provided by MOs and general practitioners, who will provide a large part of (first level) MH support. At large bases an MH Unit will be available that can provide advanced MH support. An MHU consists of a MO and a psychologist. Also, chaplains are present on most bases. In addition, ADF is supported by contracted psychologists and psychiatrists. Veteran Services provides MH support to veterans (and their families). Defense Community Organisation delivers support to ADF families.	
CAN	There is a mandatory TLD at Cyprus. It is 3 days with 2 extra days for travel. It consists of a few obligatory MH briefings and a set of educational briefings of which two have to be selected. Besides this, there are several subsidized R&R activities available.	Standard screening-process, in the form of a survey 90–180 days post-deployment. It consists of a set of standard health questionnaires (including one on PTSD symptoms) followed by an in-depth interview with an MH professional. It attempts to trace people with deployment related MH problems. Also, there is a mandatory (annual) period health assessment. Once diagnosed with an OSI an individual will be followed by one of the OTSSCs until full remission. In case there is no full remission, there is a good transition to the VAC. In-garrison MH support is covered partly by military, civilian and contracted MH professionals. All sorts of treatments are used: CBT, EMDR, medication.		After TLD, unit goes back to work for 3 half days before unit members can go on a leave. This is implemented as an additional “decompression” in order to make an optimal transition to home/base life. Besides the support from own buddies and commander, there is an Operational Stress Injury Social Support (OSISS) network, i.e., a peer support network of former operational stress injuries survivors. This is a joint activity with VAC in close collaboration with the OTSSCs.		MH care is delivered at CF Health Care Clinics across Canada. CF MH Services consists of two distinct services: Psychosocial Services and MH Services. Psychosocial Services comprise a basic level of MH care and is staffed by nurses, SWs and addictions counselors. This program is fully confidential for which no referral from a physician/MO is needed. This program is available at all clinics. MH Services consists of specialized programs such as: the OTSSC program that focuses on treatment of operational injuries, the MH program which focuses on general MH conditions and the Addiction program. For these programs a referral of a physician/MO is required. An interdisciplinary staff of psychologists, psychiatrists, MH nurses, SWs, addictions counselors and Health Services chaplains provides Service. These secondary programs are located at the larger centers.	
GBR	36 hours. 1 hour of MH briefings. Padre and psychiatric nurse on hand for informal support. All homecoming personnel see coming home DVD which is designed to protect MH (DVD MH training).	No formal screening. All personnel re-briefed/talked to 12 weeks after coming home. No formal MH care provided unless needed.		As previous box. Commanders also responsible for on-going concern about the psychological welfare of their subordinates. TRiM also available in units for informal support.		Many MH cases are handled entirely within military primary care; cases requiring formal MH input are referred to the nearest Department of Community MH site. These DsCMH provide UK-wide coverage and are staffed with a multi-disciplinary team of psychiatrists, nurses, psychologists and SWs. Referral goes via unit MOs.	
NLD	Mandatory 2 or 3 days TLD on Crete, consists of leisure activities and group discussion with MH debrief.	After 3 months: post-deployment interview with SW or chaplain After 6 months: post-deployment MH screening questionnaire Both can be followed-up by referral to MH professional, i.e., SW, psychologist, psychiatrist. Personnel can self-refer or be referred by commander to MH professional. All sorts of treatments are provided.		Commanders are responsible for MH of personnel. They can support adjustment by recuperation exercise (leisure and group discussion to provide closure of deployment) or reintegration exercise (support adjustment into new unit/with new unit members).		MO and SWs are available in garrison. They can provide support for psychosocial problems and light psychological treatment. Psychologist and psychiatrist are based in specialist MH centers providing psychological treatment. When necessary referrals to private institutions with specific treatment possibilities can be made.	
USA	No TLD is used. Decompression occurs in garrison over a 2-week period prior to units going on leave. Decompression includes screening, briefings, and education (i.e., post-deployment Battlemind training).	3–6 months post-deployment all service members undergo MH screening. Personnel can also self-refer or be command referred.		Leaders and commanders, as well as buddies have an important role in looking out for each other. This point is emphasized in the Battlemind post-deployment training. Spouses can also receive training in what to look out for.		There is organic MH support for each unit. Behavioral health clinics. Service members can also access civilian care as well.	

The columns cover the topics mission characteristics, unit characteristics, pre-deployment MH support, in-theatre MH support, post-deployment MH support. Thus, Table 1 represents the main elements of the MH protocols and current practices of the participating nations. The numbers are as per 2010.

RAAF=Royal Australian Air Force; HQ=Head Quarter; SOC=special operations command; CT=Canadian Forces; R&R=rest and recuperation; RF=regular forces; RtAPS=return to Australia Psychological Screening.

#### MH screening in service personnel

None of the partners undertook formal MH screening in the immediate pre-deployment period. However, it should be emphasized that there are extensive selection processes in selection for combat corps and most front line roles that is a form of screening. Predeployment medical assessments have normal procedures that provide a setting to detect frank disorders. Yet, new studies provided new findings for predeployment screening. First, there was no evidence for clear indicators on which screening could be based; second, the predictive value of MH screening using, for example, psychological questionnaires was not supported by evidence; and third, screening could have negative effects on the career and MH well-being of service personnel (Hyams, 2006; Rona et al., 2006). Instead, it was better to *informally* watch for signs that certain service personnel may be unfit (currently) for deployment, for instance by conversations of colleagues, commanders, chaplains or medical/MH staff. The interviews suggested that commanders and chaplains should be trained to ensure that they were aware of MH issues and should ensure that they talk to service personnel regularly.

#### MH education/training in service personnel

The importance of MH education throughout the career as well as pre-deployment MH training was generally accepted among partners and in literature (Adler et al., 2013; Mulligan et al., 2012; Mulligan, Fear, Jones, Wessely, & Greenberg, 2011). Such trained aimed to ensure MH literacy: that is, knowing how to recognize MH problems of yourself/peers/subordinates; having proper coping skills; knowing how to support others and where to go/refer for formal help if needed. However, those interviewed were aware that evidence supporting its use was sparse although it was thought that it could boost resiliency and thus work preventatively. Finally, military forces hoped the training would decrease stigma and with it the barriers to MH care. Review of the MH education and training led to observations in which: (1) giving priority to a comprehensive educational approach that uses an MH continuum model with other connected programs encompassing the whole deployment life-cycle, aimed at all ranks as well as all family members (Adler et al., 2013; Castro, Adler, McGurk, & Bliese, 2012); (2) it was felt important to not only use briefings/presentations that were passively absorbed; instead using interactive exercises with service personnel such as guided group discussions assures larger effect on service personnel; (3) integrating MH training in (stressful) operational practices makes it more tangible for service personnel to appreciate how to put the training into practice. The partners agreed that a comprehensive approach which is provided across the deployment life-cycle and integrated in operational practices ensures that MH “fitness,” like physical fitness, becomes part of daily military operations. Such an approach requires commanders and peers, as well as healthcare professionals, to be involved in the delivery of MH training and education. Some elements are better delivered by MH professionals such as guided group discussion, since they have the theoretical and practical (communicative) MH experience needed for this. However, stress-management training as part of operational tasks is thought to best be delivered by commanders or peers. Commanders and peers are better able to present a credible, maybe more technical, logistic training package because they are able to relate to the strategic and operational impact of the mission.

### Main MH practices in-theatre

#### Support provided by the unit/commander

All partners were in agreement that in the various MH educational sessions delivered during their career, service personnel needed to be taught to look after each other. MH “buddy care” was facilitated by teaching personnel to take note of signals of distress in colleagues, to be able to support to each other and, where appropriate, to encourage a colleague to discuss concerns with their commander, chaplain, MO or SW/MH nurse. In general, it was the command line that received training on recognition of MH issues, on giving advice, and adjusting leadership and referral. Furthermore, it was considered that it was a primary role of leaders to ensure that service personnel know how to access help during operations and to promote an environment where people are encouraged to access support when needed and give support to each other. This was also promoted through leadership courses advocating optimal leadership behavior and attitudes. The important and influential role of leaders on the coping of subordinates during missions has been supported by various studies (Bartone, Adler, & Vaitkus, 1998; Britt, Adler, & Bartone, 2001; Castro et al., 2012; Jones et al., 2012). A specific example of MH education aimed at a proper support system in the unit was the suicide awareness and prevention training. Such training is aimed to increase skills among personnel with respect to the detection of risk factors, providing MH first aid and suicide prevention.

#### Type of briefing after a potentially critical incident

All partners had formally abandoned single session psychological (critical incident) debriefing. This was a recent corrective measure that all partners had taken, based on the empirical evaluation of this practice in the public non-military domain that (despite the non-military setting of negative effects on MH, especially for those who were the most visibly distressed (see for a meta-analysis (van Emmerik, Kamphuis, Hulsbosch, & Emmelkamp, 2002). Amidst these developments battlemind debriefing and battlemind training are new emerging concepts (Adler, Bliese, McGurk, Hoge, & Castro, 2009). The current paradigm shared among partners is that commanders give an operational debrief after exercises and incidents, so called after “action review.” This includes that commanders, chaplains, and MH professionals practice “watchful waiting,” which implies that they try to stimulate the natural recovery processes by advocating that service personnel are experiencing normal stress reactions to an abnormal event, that normalization/readjustment is possible and expected, and that rest/food/clean clothes/getting support of unit members/calling support group home will engender this. When “battlemind psychological debriefing” was integrated into a larger “battlemind training system” the brief early interventions demonstrated to be effective with these groups. The word debriefing has been abandoned. For all partners these more *specialized* debriefings are primarily aimed at early detection and fast normalization of MH problems after experiencing trauma.

It is important to note that in case of a severe critical incident (with injuries or casualties), commanders can upscale MH support. In fact, across partners it was considered the responsibility of commanders, to decide whether a more specialized debriefing was recommended after severe incidents (Adler, Bliese, et al., 2009). All partners had protocols for these so called more “specialized” debriefings. Some partners involved an MH professional in this, to do/be present during a guided group-discussion or an educational brief. GBR typically did not involve MH professionals. Instead, they built on a new approach developed within the UK Royal Marines, known as Trauma Risk Management (TRiM) (Greenberg, Langston, & Jones, 2008). Characteristic of TRiM is that it is carried out within the unit by designated serving military personnel, who received a short training. TRiM members do work closely with the commander and MO. While this approach is very promising, there is currently a need for more evidence for its implementation (Greenberg et al., 2010; Hunt, Jones, Hastings, & Greenberg, 2013). Other countries are now also exploring to implement elements of this approach.

#### MH screening

None of the partners undertook in-theatre MH screening for the same reasons they did not undertake pre-deployment MH screening. AUS did report to conduct re-deployment screening.

#### MH team available

For all participating partners, the in-theatre MH team/unit consisted of one or more MH nurses/SWs, one or more chaplains, and an MH specialist. Some partners (CAN, US) deployed psychiatrists, but others (NLD, AUS) deployed psychologists only. The USA deployed all specialties, including psychiatrists, psychologists, social workers, and psychiatric nurses since these occupations comprise the mental healthcare team. Having an MH team or unit available in the deployment area was thought to have advantages such as minimizing barriers to care, facilitating early detection of MH problems and providing field treatment in order to keep service personnel with problems part of the unit and mission where possible. Such an approach was found beneficial for the individual and organization (see more details next section). However, it is important that MH teams/units are easy accessible. Accessibility was easier when the MH team/unit is organic to the unit compared to when there is one MH team/unit per mission area. Further, it was accepted among partners that having a multidisciplinary team available is important. Familiarity of the MH providers with the unit and the military context was felt to have a positive effect on effectiveness. And finally, having a good communication between different support providers (i.e., MO, SW, MH professionals, and commanders) was also deemed an important factor across partners. However, a MH support system was thought to require sufficient properly trained personnel to send (complete) MH teams to mission areas. If not, it was considered not advantageous to try to deploy (complete) MH teams during the whole mission, since it could compromise the quality of rear-party MH care. In this case it was thought it might be better to take a flexible approach and send out MH teams/specific specialists at major critical incidents and/or repatriate individual service personnel needing more formal MH support for treatment at home.

#### MH treatment and repatriation

The approach of frontline intervention or “forward psychiatry,” first introduced in World War I, was still practiced among partners (Jones & Wessely, 2003). This is in line with the approach adopted after potentially critical incidents (see above), since it means treating distressed personnel as quickly as possible, as close to the frontline as possible, and in many cases persuading them that their reactions area normal physiological response to the stress of battle, and that after a few days of rest, sleep, clean clothes, and hot food, he/she will be able to resume his/her military duties. This approach was substantiated by evidence showing that soldiers receiving treatment in a forward unit have lower rates of PTSD and other psychiatric symptoms, experienced less loneliness and report better social functioning compared to similarly traumatized soldiers treated in rear units (Jones, Fear, Jones, Wessely, & Greenberg, 2010; Solomon, Mikulincer, & Waysman, 1991; Solomon, Mikulincer, Waysman, & Marlowe, 1991; Solomon, Shklar, & Mikulincer, 2005). The “forward psychiatry” approach has gradually been replaced with “embedded mental health” that is organic to the unit. During WWII this was not the case, at least in the US. The embedded teams augmented the organic assets, as well as provided recuperative care, which is still “forward.” In line with this approach, all partners made efforts to treat personnel with MH issues in theatre.

There were differences in the types of treatments provided in-theatre. Some nations provided a minimal service such as psychological first aid while other nations provided a full spectrum (i.e., cognitive behavioral therapy [CBT], and eye movement desensitization and reprocessing [EMDR] and various forms of medication). Service provision was very much dependent on the type of MH providers available in-theatre for the delivery of treatment. Most partners considered it the responsibility of the commander to decide whether someone should repatriate in consultation with either the MO or the MH team. This decision was dependent on severity of illness (i.e., whether more formal/inpatient treatment is required), individual's response to treatment, specific job, and risk of staying versus risk of leaving.

### MH practices during the post-deployment phase

#### Decompression

All participating countries in this study had some sort of decompression period before service personnel could go on leave. The general definition used was that decompression is a formal way to recognize and reward the deployed troops for their experiences and begin to restore them to deploy again or return to civilian life. Decompression programs were conducted outside of and mostly immediately after leaving the theatre of operations and without family members. However, there were differences among partners in the precise context in which decompression was done. Several partners used “holiday-type” third locations (e.g., Cyprus or Crete). While it was a practice used by several partners there was no evidence yet to support its use (Jones et al., 2013). A holiday-type third location had the advantage of providing a good rest and recuperation (R&R) environment that facilitates unwinding of service personnel. On the other hand, doing decompression in a non-combat area in the country of deployment could allow better recapturing of experiences and closure of undisclosed/unresolved issues.

There were also differences among partners in the amount and type of MH sessions/elements during decompression. Generally, MH briefings and presentations were used to psycho-educate service personnel on potential issues during the adjustment at home. However, if these were not combined by guided discussions it remained unsure how service personnel perceived the messages in the briefings and presentations and whether they gained insight/skills. Research also suggested that these presentations can have a lasting effect on the mental health of service personnel (Adler, Bliese, et al., 2009; Castro et al., 2012; Mulligan et al., 2012).

Although, all participating countries had both MH providers and peers (acting on behalf of the chain of command) available, the precise role these persons played in the delivery of MH elements varied. There was consensus that both should play an important part during decompression. MH professionals could be important for their theoretical and practical MH experience while peers could serve as a better role model for proper coping (“making sense”) of deployment and they were up to date about the specific events a unit experienced during deployment. Back home, some partners sent units back to work several (half) days of what is termed “normalization,” before they could go on a leave. This was considered part of operational stress management and, like decompression, had the purpose of not losing sight of each other immediately, and detection and addressing of potential adjustment problems.

#### Follow-up by MH professionals

The type and length of MH follow-up varied among partners. Only GBR had no formal MH follow-up post-deployment although it was currently engaged on a randomized controlled trial of post deployment screening. The relatively low prevalence of PTSD, as established by the health surveillance research conducted by the KCMHR, together with the low specificity of PTSD screening measures, was used as rationale for not undertaking screening pre-, during or post-deployment (KCMHR 10 year report). Long-term detection of operational stress injury was considered the responsibility of the individual serviceman, commanders, colleagues, and family. GBR did have routine, periodic, and special medical examinations of individual's known to have returned from an operational deployment. MOs were instructed to be alert for signals of psychological injury. Screening measures in MO/GP settings had been shown to improve rates of detection and outcomes, so there was potential to question this non-interventionist approach.

The other partners did use some form of MH follow-up post-deployment, usually between 3 and 6 months after return. The procedures differed however. Several studies showed that the impact of combat can be severe and long lasting and often follows a complex course (Solomon, Shklar, Singer, & Mikulincer, 2006). While delayed-onset PTSD (i.e., the development of PTSD more than 6 months post-trauma) is generally characterized by partial or subsyndromal diagnoses within the first 6 months, there are individuals who develop PTSD after more than 6 months who do not meet the criteria for partial or subsyndromal PTSD before that (Andrews, Brewin, Philpott, & Stewart, 2007; Carty, O'Donnell, & Creamer, 2006; Goodwin et al., 2012; Solomon & Mikulincer, 2006). There is a percentage showing exacerbations or reactivations of prior symptoms after more than 6 months. Given that over 20% of individuals who develop PTSD have the delayed form, there is evidence of the need to undertake longer follow-up than 6 months to detect delayed, exacerbated or reactivated PTSD symptoms in annual medical assessments (Horesh, Solomon, Zerach, & Ein-Dor, 2011; Smid, Kleber, Rademaker, van Zuiden, & Vermetten, 2013; Smid, Mooren, van der Mast, Gersons, & Kleber, 2009). A further benefit of screening is that it makes mental health questions more familiar and introduces military personnel to direct contact with a mental health provider.

Modern warfare is characterized by a “new” weapon, that is, the IED with a additional ‘signature wound’, which is (m)TBI. The interest for this blast related (m)TBI as was introduced by the US was also reflected by an enormous popularity for this new disorder and its treatment. A recent Medline-evaluation by Wallace (2009) from 2001–2008 substantiated that IED-related (m)TBI can not be ignored as one of the most important injuries associated with current military missions. There is high overlap with symptoms of PTSD, which contributed to strong debates about diagnosis (Creamer, O'Donnell, & Pattison, 2005; Ruff, Riechers, & Ruff, 2010; Vanderploeg, Belanger, & Curtiss, 2009), symptom trajectory (Bryant, O'Donnell, Creamer, McFarlane, & Silove, 2013), and optimal treatment (Davis, Walter, Chard, Parkinson, & Houston, 2013). In our review material, there was an increased focus across partners on proper detection and treatment of soldiers having obtained (m)TBI due to blasts of IEDs during their deployment and recognition for multidisciplinary collaborative care models of treatment in primary care to collectively address the full spectrum of postwar physical and neurocognitive health concerns (Wilk, Herrell, Wynn, Riviere, & Hoge, 2012). This was accomplished through a combination of research, educational programs, and policy development.

With respect to the MH support infrastructure available, it can be concluded that all partners had multiple services in place for rear-party MH support. Generally, first-line MH support was delivered by MOs and MH nurses/SWs, who are usually available at local bases. For more formal (second-line) case management all partners had specialized clinics/centers available having multidisciplinary MH teams. Formal MH support was delivered by psychologists or psychiatrists and consists of a wide spectrum of treatments. For PTSD, general CBT and EMDR were the standard treatments. However, medication was sometimes also given. Further, all partners had services (programs) in place for addressing other problems such as AD, alcohol/drug abuse, depression, and suicide. Although there was an effort to have MH support delivered primarily by uniformed MH professionals, both contracted and/or standard civil MH services are relied on to some extent by all partners.

#### Follow-up and care by unit/commander

None of the partners had standardized follow-up by the unit or commander after a mission. However, it was acknowledged that buddies and leaders have an important role in detecting of MH issues, facilitating natural recovery (making sense and proper coping) after an intense deployment, giving support/advise and guiding peers/subordinates to formal support if needed. Some partners offered commanders the opportunity to implement a non-arduous enjoyable military exercise combined with psycho-education and/or group discussion to address these issues. Proper dealing with these issues was also stimulated by MH education and pre-deployment training packages. For example, there was the “Battlemind training” (Adler, Castro, & McGurk, 2009; Castro et al., 2012) introduced by the USA that has the objective to mentally prepare soldiers for the rigors of combat and other aspects of military deployments, to assist them in their successful transition back home and to provide the skills to assist their “Battle Buddy” in the transition to home. This type of training became popular among partners (see, e.g., “BattleSMART” training of AUS and “Road to Mental Readiness” training of CAN). If there was a proper climate (no stigma and proper MH knowledge and skills available) there would be no need for standardized follow-up by the commander/unit, as the unit was considered a natural support system. Yet, this may be too idealistic as some reports suggest (Hoge et al., 2004). Most partners had some sort of peer support groups/networks in place. The opinion was that good peer support, with trained peers liaising with MH professionals, was found crucial in a good support system (Keller et al., 2005; Pfeiffer et al., 2012). As it is rooted in the military context, it is thought to be helpful in diminishing the remaining stigma around having MH issues and offers a lower barrier to care. However, psychoeducation, resilience training, and the effectiveness of peer support systems still are to be supported by rigorous evidence, despite the broad acceptance of these roles (Greenberg, Langston, Iversen, & Wessely, 2011).

### Common bottlenecks for military MH care

Military MH care has come a long way and has reached an established status that more than ever meets the criteria for state of the art services. Nevertheless, several common bottlenecks in current practices are worthwhile to discuss, since from this discussion promising future developments can be inferred that may lead to more effective military MH care, assuring its state of the art-status. Main common bottlenecks/needs and suggested promising developments will be discussed.

#### Barriers to MH care

The first and most important common bottleneck for effective MH support was the barrier to MH care. This was partly explained by the fact that there is still a stigma around experiencing MH issues during/after deployment among serving personnel that prevents them from seeking treatment (Gould et al., 2010; Hoge et al., 2004). Military organizations encourage self-reliance and resilience, appropriately to the nature of the task of service personnel. Experiencing MH problems was often seen as a failure of self-reliance and was associated with shame and guilt (Greene-Shortridge, Britt, & Castro, 2007; Kim, Thomas, Wilk, Castro, & Hoge, 2010; Pietrzak et al., 2009). Admitting to an MH disorder was viewed as a cause of disapproval from peers. Also, service personnel were reported to be afraid of the negative effects it may have on their career in the military. Another critical factor was the fact that counseling (such as CBT) relied heavily on verbal skills. Many service personnel would not find this very attractive or even fearful, because they were not used to talking about problems, instead they are often more action-oriented. This aspect of counseling might therefore hinder service personnel from seeking MH support, prompt them to terminate their treatment prematurely or render it less effective. This asks for new methods/tools that go around talking about MH problems, with a positive resilience approach and that better fit military context as this would all lower the barrier to care.

#### Availability of MH care providers

The second common bottleneck for effective MH support was an insufficient availability of MH care providers. This was partly explained by the fact that in missions such as the current one in Afghanistan, MH care personnel is highly dispersed due to the geography of the country. Another aspect was a simple shortage in MH professionals, especially uniformed psychiatrists and psychologists. These professionals were difficult to recruit and keep. Related to this is the finding that primary care level was sometimes inefficient, because MOs, GPs, and SWs lacked specific clinical training and skills. This asks for new tools that focus on self-empowerment of service personnel, that is, that train service personnel in how to recognize and normalize MH problems by themselves/in the unit as this would lower the dependency on the scarce MH care providers.

#### Correspondence between the MH support system and deployment life-cycle

An optimal Military MH support system needs to have a seamless correspondence with the cyclic character of deployments. This implies: (1) adequate mental resiliency building training pre-deployment; (2) MH support focusing on fast normalization in-theatre and during decompression; and (3) adequate MH follow-up post-deployment. Together this was hoped to lower the chance that service personnel will experience MH complaints or that MH complaints develop into full-blown MH disorders. All partners already started working with an MH continuum model with connected programs and practices encompassing the whole deployment life-cycle. However, there is still room for optimization of the connection between current MH programs/practices and an efficient application in each deployment phase. Also, there is still room for new tools optimally suiting an MH continuum model.

#### Providing an “armor for your mind” that helps service personnel to take control over stress

It was expressed that advancement of training packages for service personnel focused on the promotion of stress resiliency and attaining control over stress reactions was needed. Such training packages are already used to some extent by all partners, for example the Battlemind training of USA, the BattleSMART training of AUS and the Road to Mental Readiness training of CAN. However, there is still room for extension and innovation of these packages. The largest of these initiatives is the Army's Comprehensive Soldier Fitness (CSF) program, which has been disseminated to more than 1 million soldiers. However, to date, CSF has not been independently and objectively reviewed, and the degree to which it successfully promotes adaptive outcomes and prevents the development of deployment-related mental health disorders such as PTSD is still uncertain (Peterson, Park, & Castro, 2011; Seligman & Fowler, 2011; Steenkamp, Nash, & Litz, 2013). Important elements in these packages are teaching of human stress reactions and stress normalization mechanisms, how to recognize stress reactions in themselves, and to mitigate the impact of stress reactions, that is, gaining control over stress. There is also a request for new methods/tools with a positive resilience approach, that go around talking about MH issues, that are self-empowering and that comply with military context to be applied in an MH continuum model.

#### Social leadership training

There was a growing acknowledgement that MH support is an important part of daily military operations and that leaders and commanders play a pivotal role in this throughout the deployment-life cycle. There is ample evidence that the person characteristics of military leaders play a critical role in the resiliency of military personnel and the risk in development of MH complaints (Adler et al., 2008; Britt, Davison, Bliese, & Castro, 2004; Britt, Wright, & Moore, 2012; Davidovitz, Mikulincer, Shaver, Izsak, & Popper, 2007; Iversen et al., 2008; Johnson, Grasso, & Maslowski, 2010). Leaders have the power to influence the motivation, thinking, and coping behavior of service personnel (Davidovitz et al., 2007; Jones et al., 2012). Therefore, coaching junior leaders in social leadership can serve as an important preventative effort. Also, as leaders often serve as role models it has the potential to diminish the remaining stigma. Moreover, it also can lower the burden on the scarce MH care providers. The ways to foster proper leadership attitude and skill was through the teaching of being a role model (leading figure), facilitate open communication in the unit, discuss “lessons learned” after incidents/mistakes (facilitation of sense making). Also creating meaningful and challenging tasks, monitor the fulfillment of basic needs, including rest and leisure activities (keeping the unit physically fit), and lastly, encourage unit members to use the stress control strategies that are most appropriate for them.

#### Training peer counseling across all levels

A promising development that was seen was training peer counseling across all levels. That is, training peers in how to recognize MH issues in colleagues and how to help colleagues cope with MH issues. This type of training can work preventatively as it may facilitate faster tackling of MH issues within the unit, thereby preventing that these develop into more serious MH complaints. This lowers the dependency on MH professionals. Being rooted in military context, a peer support system has the potential to change culture, and in particular to make it more acceptable for military personnel to admit to psychological distress when they experience it, and to present for treatment when they need it. Most of the partners were already working with peer support systems (e.g., the U.S. Army's Comprehensive Soldier Fitness, TRiM peers, collegial networkers, the peer support coordinator of the Operational Stress Injury network, Battle buddies), but there is room for improvement. Ways to advance these systems are thought to include: (1) Training of more peer counselors; (2) Wider/more efficient administration of the peer support system throughout the deployment life-cycle, and (3) Improvement of coordination between trained peers, command line, primary care level, and MH professionals (i.e., better communication and clearer roles between them).

## Summary and conclusion

This paper described the results of a comparative analysis of five NATO partner countries (AUS, CAN, GBR, NLD, and USA) with respect to their protocols and current practices of MH support before, during, and after operational deployment. The evaluation focused on prevention, intervention, and treatment. The complete chain of MH support was taken into account. Part of this analysis was to compare existing MH protocols and current practices, several common bottlenecks for effective military MH support as well as important developments.

Our interviews and document-analysis revealed that each of the participating organizations has many initiatives to support the MH of service personnel in the different phases of the deployment life-cycle. Key elements were identified: (1) awareness campaigns directed at overcoming the stigma associated with experiencing MH issues; (2) buddy training and teaching service personnel how to help their battle buddies meet and overcome any MH challenges that they may encounter (e.g., GBR's TRiM program); (3) specific pre-deployment training packages for service personnel focused on the promotion of stress resiliency and attaining control over stress reactions (e.g., the Battlemind training of the USA, the BattleSMART training of AUS and the Road to Mental Readiness training of CAN); (4) peer-support networks with former operational stress injury survivors acting as speakers and counselors; (5) assistance programs that are available 24 hours a day, 7 days a week for confidential, short-term counseling; (6) addiction and suicide programs focused on creating awareness and preventative efforts; (7) support programs for family members and other close ones; (8) in-theatre multidisciplinary MH support teams; and (9) (holiday-type) decompression with psycho-education and R&R.

Our cross comparison showed that the different organizations adopted many similar MH protocols and practices. Also, all strived to use evidence- or evaluation-based protocols and practices. For example, none of the participating partners undertook formal MH screening pre-deployment or in-theatre as there was no evidence for clear indicators on which screening can be based and the predictive value of MH screening using, for example, psychological questionnaires is not supported by evidence. Additionally, all partners formally abandoned single session psychological (critical incident) debriefing. This was a recent corrective effort that all partners undertook, based on the empirical evaluation of this practice demonstrating no evidence of its effectiveness and even risks of negative effects on MH, especially for those who are the most visibly distressed. Instead, all partners made an effort to educate, and train, service personnel throughout their career as well as pre-deployment trainings about MH and stress management. Battlemind debriefing and battlemind training are new emerging concepts. Education of all partners was focused on: knowing how to recognize MH problems of yourself/peers/subordinates; having proper coping skills; knowing how to support others and where to go/refer for formal help if needed. End-goals were boosting stress resiliency and creating a proper support system in the unit. In-theatre, all participating organizations took an MH care approach of watchful waiting and of frontline intervention/forward psychiatry. This meant that all organizations aimed to treat a stressed serviceperson as quickly as possible, as close to the frontline as possible, and doing everything to persuade him/her that his is a normal physiological response to the stress of battle, and that after a few days of rest, sleep, clean clothes, and hot food, he/she will be able to resume his/her military duties. In order to do this, all partners have an in-theatre MH team/unit consisting of one or more MH nurses/SWs, one or more chaplains, and an MH specialist. Finally, all participating organizations had some sort of decompression period before service personnel could go on leave to acknowledge and reward the deployed troops for their efforts and begin to prepare them to deploy again or return to civilian life. When further care was requested all partners had an MH support infrastructure in place. There are no methodologically sound studies comparing different types or lengths of MH follow-up. Nevertheless, it may be advised that if follow-up is done, it is best to: (1) use multiple validated MH questionnaires; (2) incorporate an individual interview with an MH professional/examiner; and (3) plan follow-up at different time intervals.

In conclusion, the historical evolution of military MH care has been considerable and has now reached an established status that more than even needs to meet the criteria of state of the art service. Nevertheless, some common bottlenecks in current MH practices could be identified including the remaining stigma about mental illness among service personnel, and sufficient availability of MH professionals in theatre. Therefore, across military organizations a consensus exists about the importance of delivering MH programs (1) with a positive resilience approach; (2) integrated in daily military operations; (3) focused on self-regulation (self-empowering of service personnel); (4) executed and adhered to by peers and commanders; and (5) as part of an MH continuum model. Promising current military MH care developments include social leadership training and training peer counseling across all levels. Both facilitate faster tackling of MH issues within the unit, which lowers the dependency on the scarce MH professionals. Also, being rooted in military context, both can help diminish the remaining stigma.

The results of this analysis can be used to develop new policies and practices that strengthen the military MH care that the participating organizations currently provide in order to sustain a good work environment, operational effectiveness and MH well-being of their service personnel. Furthermore, the present results can be used to develop an even more efficient collaboration between partners in their mutual MH care efforts, whereby they will be better able to face the challenges of current and future military missions.
